# Association of abdominal adiposity, hepatic shear stiffness with subclinical left-ventricular remodeling evaluated by magnetic resonance in adults free of overt cardiovascular diseases: a prospective study

**DOI:** 10.1186/s12933-023-01828-1

**Published:** 2023-04-29

**Authors:** Yali Qu, Jing Liu, Jing Li, Sumin Shen, Xiaoyi Chen, Hehan Tang, Yuan Yuan, Chunchao Xia, Liping Deng, Guoyong Chen, Tianying Zheng, Jie Chen, Lisha Nie, Fang Yuan, Nanwei Tong, Liqing Peng, Bin Song

**Affiliations:** 1 Department of Radiology, West China Hospital of Sichuan University, Sichuan Chengdu, China; 2 Department of Endocrinology and Metabolism, West China Hospital of Sichuan University, Chengdu, Sichuan China; 3 GE Healthcare, MR Research China, Beijing, China; 4 Department of Radiology, Sanya People’s Hospital, Hainan Sanya, China

**Keywords:** Magnetic resonance imaging, Ectopic fat deposition, Visceral adipose tissue, Ventricular remodeling, Obesity, Metabolic syndrome

## Abstract

**Background:**

Abdominal ectopic fat deposition and excess visceral fat depots in obesity may be related to cardiovascular disease (CVD) as both are involved in the metabolic syndrome (MetS). The awareness of the link between abdominal adiposity and subclinical cardiac remodeling would help improve treatment and outcome. Besides, liver fibrosis has also shown a potential relationship with cardiac dysfunction. Thus, we aimed to investigate the associations of magnetic resonance (MR)-based abdominal adiposity and hepatic shear stiffness with subclinical left ventricular (LV) remodeling while taking account of MetS-related confounders in adults free of overt CVD.

**Methods:**

This was an exploratory, prospective study of 88 adults (46 subjects with obesity, 42 healthy controls) who underwent 3 T cardiac and body MR exams. Measures of abdominal MR included hepatic and pancreatic proton density fat fraction (H-PDFF and P-PDFF), hepatic shear stiffness by MR elastography, and subcutaneous and visceral adipose tissue (SAT and VAT). Cardiac measures included epicardial adipose tissue (EAT) and parameters of LV geometry and function. Associations were assessed using Pearson correlation and multivariable linear regression analyses, in which age, sex, and MetS-related confounders were adjusted for.

**Results:**

The LV ejection fractions of all participants were within the normal range. Higher H-PDFF, P-PDFF, SAT and VAT were independently associated with lower LV global myocardial strain parameters (radial, circumferential and longitudinal peak strain [PS], longitudinal peak systolic strain rate and diastolic strain rate) (β = − 0.001 to − 0.41, *p* < 0.05), and P-PDFF, SAT and VAT were independently and positively associated with LV end-diastolic volume and stroke volume (β = 0.09 to 3.08, *p* ≤ 0.02) in the over-all cohort. In the obesity subgroup, higher P-PDFF and VAT were independently associated with lower circumferential and longitudinal PS, respectively (β = − 0.29 to − 0.05, *p* ≤ 0.01). No independent correlation between hepatic shear stiffness and EAT or LV remodeling was found (all *p* ≥ 0.05).

**Conclusions:**

Ectopic fat depositions in the liver and pancreas, and excess abdominal adipose tissue pose a risk of subclinical LV remodeling beyond MetS-related CVD risk factors in adults without overt CVD. VAT may play a more considerable role as a risk factor for subclinical LV dysfunction than does SAT in individuals with obesity. The underlying mechanisms of these associations and their longitudinal clinical implications need further investigation.

**Supplementary Information:**

The online version contains supplementary material available at 10.1186/s12933-023-01828-1.

## Background

Obesity has been a pandemic, critical and costly disease worldwide for decades. Body mass index (BMI) is commonly used to identify obesity with different cutoff values across various racial or ethnic groups. For example, the BMI cutoff for obesity among adults is 30 kg/m^2^ in Western countries and 27.5 kg/m^2^ in Asia populations [1, 2]. The global prevalence of obesity has nearly tripled since 1975 and continues to grow [3]. In China, the prevalence of obesity (BMI ≥ 27.5 kg/m^2^) among adults is about 12.9% with the highest proportion in western region (13.2%) [1].

In obesity, the overload of calories gives rise to fat accumulation in the visceral depots (e.g., intra-abdominal and epicardial adipose tissue [EAT]) and ectopic fat deposition (e.g., liver and pancreas), which is highly involved in the development of metabolic disorders [4, 5]. It has been shown that the leading adverse consequence of metabolic syndrome (MetS) is cardiovascular disease (CVD) [6].

The underlying mechanisms of the relationship between abdominal adiposity and CVD are complicated and less well understood. One of the putative mechanisms is that ectopic fat deposition and excess visceral adipose tissue (VAT) release fat-derived toxic metabolites and activate inflammatory pathways triggering a cluster of pathophysiological changes that promote the development of CVD [7]. The development of CVD in obesity is a gradual change, and subclinical impairment of cardiac function occurs before overt clinical manifestations [6]. Subclinical cardiac alterations might be reversible; thus, awareness of the link between abdominal adiposity and subclinical cardiac remodeling would help improve treatment and outcome.

Cardiac magnetic resonance (MR) has been a vital modality for the highly accurate and reproducible assessment of cardiac geometry and function, especially for the evaluation of subclinical dysfunction using myocardial strain parameters [8]. Abdominal MR allows for the accurate and simultaneous quantification of hepatic and pancreatic steatosis as well as adipose tissue area. Limited data with differences in cohort composition and cardiac MR indices, are available to support the associations of MR-based hepatic steatosis, subcutaneous adipose tissue (SAT) and VAT with subclinical left ventricular (LV) remodeling [9–12]. For example, one study reported that in nondiabetic men with nonalcoholic fatty liver disease, hepatic steatosis and VAT were inversely correlated with peak filling rate [12]; another study showed that in subjects with type 2 diabetes and healthy controls, hepatic steatosis correlated negatively with LV peak systolic strain and diastolic strain rate, while without adjusting for potential confounders, such as MetS-related CVD risk factors [11]. Given that ectopic fat deposition and CVD have shared risk factors (i.e., components of MetS), one way to better explore whether the association of abdominal adiposity with subclinical cardiac remodeling exists independently of MetS would be to regard MetS-related factors as potential confounders.

In addition, liver fibrosis in chronic liver disease has shown a potential relationship with cardiac dysfunction [13]. A few studies observed that liver fibrosis estimated by histology or MR elastography (MRE) was associated with increased epicardial fat [14, 15]; however, limited information exists on the association between liver fibrosis and MR-based cardiac alteration.

To this end, the purpose of this exploratory study was to investigate the associations of MR-based abdominal adiposity and hepatic shear stiffness with subclinical LV remodeling while taking account of MetS-related confounders in adults free of overt CVD.

## Methods

### Study design

This was a prospective, cross-sectional, single-center analysis. Adults with obesity and healthy controls were consecutively recruited between January 2020 and May 2022 through advertising. Inclusion criteria included age ≥ 18 years; body mass index (BMI) ≥ 27.5 kg/m^2^ for subjects with obesity, BMI < 23 kg/m^2^ for healthy controls (definition for Asian populations [1, 2]. Exclusion criteria were viral hepatitis; autoimmune hepatitis; hepatotoxic medications; a history of cardiovascular diseases or any cardiovascular procedures, endocrine diseases (e.g., hyperthyroidism and hypothyroidism); major systemic diseases affecting the myocardium; metabolic diseases; obstructive sleep apnea; contraindication(s) to MR examination; or pregnancy or trying to become pregnant.

This study was approved by our Institutional Review Board. Informed written consent was obtained from each subject.

### Demographic, anthropometric, and laboratory data

Demographic, anthropometric, and laboratory data of all subjects were collected, including age, sex, weight, height, BMI, waist circumference, hip circumference, heart rate, systolic and diastolic blood pressure (BP), hypertension, fasting and 2 h serum glucose concentration, prediabetes, and serum lipid profiles (total cholesterol [TC], triglycerides [TG], high-density lipoprotein cholesterol [HDL-C], and low-density lipoprotein [LDL-C]). Heart rate was assessed using a 12-lead electrocardiogram (ECG). BP was measured twice in the sitting position with feet on the floor and back supported after 15 min of rest to help better reduce the white coat effect, and the mean BP values were calculated. Hypertension was defined by systolic BP ≥ 130 mmHg and/or diastolic BP ≥ 80 mmHg [16]. Serum glucose concentrations were measured using the oral glucose tolerance test (OGTT). Prediabetes was diagnosed if fasting blood glucose was 5.6–6.9 mmol/L or 2 h blood glucose was 7.8–11.0 mmol/L [17]. The MetS was defined according to the Harmonization definition, i.e., a diagnosis of the MetS is made when any 3 of the 5 following risk factors are present: (1) waist circumference ≥ 88 cm for women and ≥ 102 cm for men, (2) TG ≥ 1.7 mmol/L, (3) HDL-C < 1.3 mmol/L in women and < 1.0 mmol/L in men, (4) systolic BP ≥ 130 mm Hg and/or diastolic BP ≥ 85 mm Hg, and (5) fasting glucose level ≥ 5.6 mmol/L [18].

### MR examinations

Non-contrast cardiac and abdominal MR examinations were performed at 3 T (MAGNETOM Skyra, Siemens Healthcare for cardiac MR; Discovery MR750W, GE Healthcare for abdominal MR) on the same day. To minimize potential physiological confounding factors, participants were instructed to fast for a minimum of 4 h before abdominal scanning. Subjects were scanned in the supine position with an 18-channel phased-array body coil for cardiac imaging and with an 8-channel torso phased-array receive coil for abdominal imaging. A dielectric pad was placed between the surface coil and the abdominal wall to reduce shading from B1 heterogeneity. MRE examinations were performed using a 60-Hz paddle vibration frequency, as previously described [19].

### Cardiac MR sequences and analysis

With a standard ECG-triggering device, data were acquired during the end-expiratory breath-hold period. A segmented breath-hold balanced steady-state free precession (bSSFP) sequence was used to obtain 8–14 continuous cine images from the heart base to the apex in the short-axis view, and LV two- and four-chamber cine images in the long-axis view. Twenty-five phases were reconstructed in a cardiac cycle. The temporal resolution was 39.34 ms. Other acquisition parameters are summarized in Table 1.

**Table 1:** Parameters for MR Techniques

Acquisition parameters	bSSFP	MRI-PDFF	2D MRE	LAVA-Flex
TR (ms)	3.3	7.3	1000	4.5
TE (ms)	1.2	0.97, 1.78, 2.59, 3.40, 4.21, 5.02	Min full	1.3, 2.7
FA (degrees)	41	3	90	15
Slice thickness (mm)	8	7	8	5
Number of slices	8–14	36	4	52
Intersection gap (mm)	0	0	2	0
Matrix	208 × 166	160 × 160	80 × 80	300 × 256
FOV (cm)	36 × 32	50 × 40	50 × 50	50 × 40
BW (kHz)	1145	± 111	± 250	± 143
NEX	1	0.5	1	1

*2D* two-dimensional, *bSSFP* balanced steady-state free precession, *BW* bandwidth; *FA* flip angle, *FOV* field of view, *LAVA-Flex* liver acquisition with volume acceleration flex, *MRE* magnetic resonance elastography, *MRI-PDFF* magnetic resonance imaging-proton density fat fraction, *NEX* number of excitations, *TR* repetition tim, *TE* echo time

EAT volume, LV geometry, and global systolic function were measured on the short-axis cine images; LV global myocardial strain was assessed on the long-axis two- and four-chamber and short-axis cine images. Parameters of LV geometry and global systolic function included LV mass (LVM) at end-diastole, mean LV regional values for 16 myocardial segment thicknesses (excluding the apex) (LVMT), LV ejection fraction (LVEF), LV end-diastolic volume (LVEDV), LV end-systolic volume (LVESV), and stroke volume (SV). LV global myocardial strain parameters included radial, circumferential, and longitudinal peak strain (R-PS, C-PS and L-PS), peak systolic strain rate (R-PSSR, C-PSSR and L-PSSR), peak diastolic strain rate (R-PDSR, C-PDSR and L-PDSR). If applied to C-PS, C-PSSR, L-PS, L-PSSR, and R-PDSR, the values are negative. A lower absolute value for myocardial strain parameters means worse myocardial contractility. Analysis details are described in the Additional file 1.

Two experienced cardiovascular radiologists (with more than 3 years of experience) who were blinded to the clinical and abdominal MR data performed the cardiac image analyses using CVI42 v5.11.3 (Circle Cardiovascular Imaging, Calgary, Canada). The intra- and interobserver variability of the LV global myocardial strain parameters were assessed (details in the Additional file 1).

### Abdominal MR sequences and analysis

Hepatic and pancreatic proton density fat fractions (H-PDFF and P-PDFF) were acquired using iterative decomposition of water and fat with echo asymmetry and least-squares estimation quantitation (IDEAL-IQ) sequence in one 20-s breath-hold. Abdominal SAT and VAT area were measured on axial fat images generated by axial liver acquisition with volume acceleration flex (LAVA-Flex) sequence. Hepatic shear stiffness was generated by breath-hold 2D 60 Hz MRE using spin-echo echo-planar imaging (SE-EPI). Acquisition parameters are listed in Table 1. The analysis of H-PDFF, P-PDFF, SAT, VAT, and hepatic shear stiffness is further described in the Additional file 1.

Two experienced abdominal radiologists (with more than 4 years of experience) who were blinded to the clinical and cardiac MR data performed abdominal image analyses. H-PDFF, P-PDFF and hepatic shear stiffness analyses were performed using Horos imaging software (Horos Project, Geneva, Switzerland), and SAT and VAT areas were analyzed using sliceOmatic v5.0 (TomoVision, Magog, Canada). The intra- and interobserver variability of abdominal MR measures were assessed (details in the Additional file 1).

### Statistical analyses

Demographic, anthropometric, and laboratory data and MR outcomes were summarized descriptively. Continuous variables were expressed as mean ± standard deviation or median with interquartile range, and categorical variables were expressed as number and percentage. For the negative values of LV global myocardial strain parameters, the absolute values were used for the statistical analyses. All variables were compared between subjects with obesity and healthy controls using Mann–Whitney U tests or chi-squared tests of proportions. The intraclass correlation coefficient (ICC) was calculated to assess the intra- and interobserver variability.

In univariate analyses, Pearson correlation coefficients (r) were calculated to explore the associations of H-PDFF, P-PDFF, SAT, VAT and hepatic shear stiffness with cardiac measures in the over-all cohort, in the obesity subgroup, and in the control subgroup, respectively. Relationships that showed significant differences in univariate analyses were then further examined using multivariable linear regression analyses. In each regression, the outcome was one cardiac MR measure, and the predictors consisted of one abdominal MR measure as well as the covariates of (a) age, sex, and with/without MetS (Model 1), or (b) age, sex, hypertension, TG, HDL-C, and prediabetes (Model 2). MetS, hypertension and prediabetes were not adjusted in healthy controls, as no subject had any of them. A 2-tailed p value < 0.05 was considered statistically significant. All analyses were conducted with SPSS Version 26.0 (IBM, Armonk, NY, USA). As this was an exploratory study, correction for multiple comparisons was not applied.

## Results

### Cohort characteristics

**Fig. 1:**
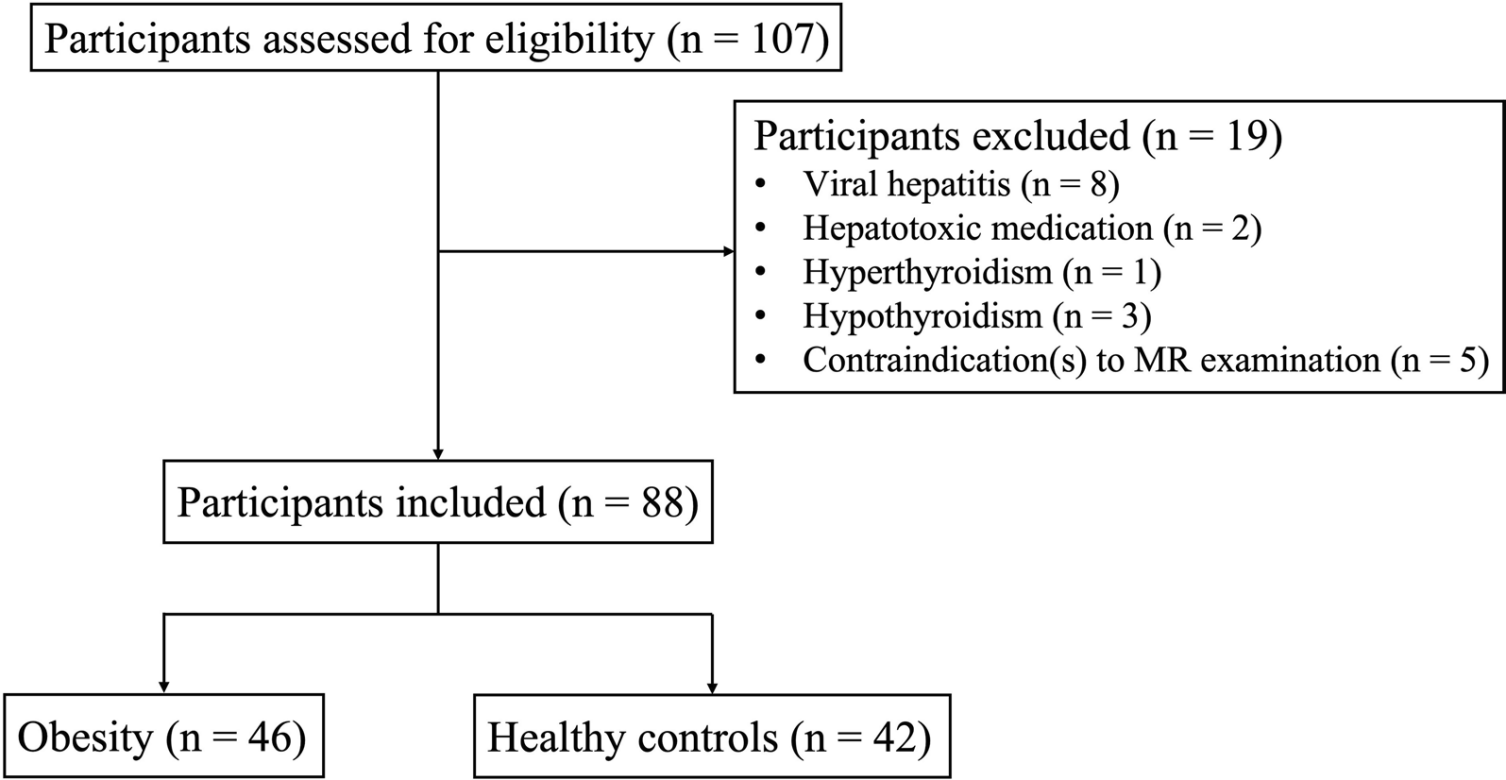
Flow chart of participant inclusion and exclusion

Of 107 participants assessed for eligibility, 88 adults were recruited for this study (Fig. 1). Of the 88 subjects (age 30.1 ± 7.5 years, 43.2% females, median BMI 27.7 kg/m^2^), 46 subjects (age 32.0 ± 7.5 years, 43.5% females, BMI 30.4 ± 1.9 kg/m^2^) were with obesity, and 42 subjects (age 29.7 ± 7.4 years, 42.9% females, BMI 20.4 ± 1.4 kg/m^2^) were healthy controls. Subjects with obesity and healthy controls did not differ in age (*p* = 0.09) or sex (*p* = 0.95) (Table 2). Twenty-seven subjects with obesity (27/46 = 58.7%) were diagnosed with hypertension, and 20 subjects with obesity (20/46 = 43.5%) had prediabetes. Nineteen subjects with obesity (19/46 = 41.3%) were diagnosed with MetS. The LVEF values of all subjects were within the normal range. Demographics, anthropometry, laboratory, and MR measures are summarized in Table 2.

**Table 2:** Demographics, Anthropometry, Laboratory and MR measures of Study Cohort

Characteristic	All (n = 88)	Obesity (n = 46)	Control (n = 42)	*p*
Demographic and anthropometric data				
Age, *years*, mean ± SD (range)	30.1 ± 7.5 (20–51)	32.0 ± 7.5 (20–51)	29.7 ± 7.4 (21–51)	0.09
Sex (female), n (%)	38 (43.2)	20 (43.5)	18 (42.9)	0.95
BMI, *kg/m*^*2*^, median (IQR)/ mean ± SD	27.7 (9.9)	30.4 ± 1.9	20.4 ± 1.4	< 0.001*
Waist circumference, *cm,* mean ± SD	87.3 ± 15.8	99.7 ± 11.5	73.8 ± 5.0	< 0.001*
Hip circumference, *cm*, mean ± SD	100.3 ± 8.4	107.2 ± 4.1	92.8 ± 4.2	< 0.001*
Heart rate, *b.p.m*, mean ± SD	75.8 ± 8.2	77.3 ± 8.2	74.3 ± 8.1	0.09
Systolic BP, *mmHg*, mean ± SD	116.7 ± 13.2	124.0 ± 9.7	108.7 ± 11.8	< 0.001*
Diastolic BP, *mmHg*, mean ± SD	75.6 ± 8.6	79.2 ± 6.5	71.6 ± 8.9	< 0.001*
Laboratory, median (IQR)				
AST, *U/L*	20.0 (8.8)	21.5 (10.8)	18.5 (8.0)	0.06
ALT, *U/L*	20.5 (20.0)	30.5 (28.5)	14 (9.3)	< 0.001*
TC, *mmol/L*	4.3 (1.4)	4.6 (1.6)	4.0 (1.0)	0.001*
TG, *mmol/L*	0.8 (1.3)	1.6 (2.6)	0.5 (0.3)	< 0.001*
HDL-C, *mmol/L*	1.4 (0.5)	1.2 (1.5)	1.6 (0.4)	< 0.001*
LDL-C, *mmol/L*	2.3 (1.1)	2.5 (3.4)	1.9 (0.8)	0.001*
Fasting serum glucose, *mmol/L*	5.0 (0.8)	5.5 (0.8)	4.8 (0.4)	< 0.001*
2-h serum glucose, *mmol/L*	5.0 (2.1)	6.2 (2.4)	4.5 (0.7)	< 0.001*
Hypertension, n (%)	27 (30.7)	27 (58.7)	0 (0.0)	NA
Prediabetes, n (%)	20 (22.7)	20 (43.5)	0 (0.0)	NA
MetS, n (%)	19 (21.6)	19 (41.3)	0 (0.0)	NA
MR-based Cardiac measures, mean ± SD				
EAT, *cm*^*3*^	34.3 ± 18.3	47.9 ± 14.6	19.4 ± 6.9	< 0.001*
LVM, g	84.1 ± 19.5	90.6 ± 19.6	77.1 ± 17.0	0.002*
LVMT, *mm*	5.9 ± 0.8	5.97 ± 0.83	5.77 ± 0.71	0.20
LVEF, *%,* (range)	61.7 ± 4.9 (50.0–71.7)	62.7 ± 4.7 (54.7–71.7)	60.6 ± 5.0(50.0–69.8)	0.10
LVEDV, *mL*	144.8 ± 28.5	159.1 ± 27.2	129.1 ± 20.7	< 0.001*
LVESV, *mL*	56.0 ± 12.5	59.9 ± 13.5	51.6 ± 9.7	0.01*
SV, *mL*	88.8 ± 19.9	99.2 ± 17.7	77.4 ± 15.6	< 0.001*
R-PS, *%*	33.6 ± 5.4	32.1 ± 5.0	35.2 ± 5.5	0.02*
C-PS, *%*	− 20.0 ± 2.0	− 19.6 ± 1.9	− 20.4 ± 2.0	0.047*
L-PS, *%*	− 14.5 ± 2.7	− 13.3 ± 2.8	− 15.8 ± 2.0	< 0.001*
R-PSSR, *s*^*−1*^	1.99 ± 0.72	1.86 ± 0.43	2.15 ± 0.92	0.10
C-PSSR,* s*^*−1*^	− 1.04 ± 0.17	− 1.01 ± 0.12	− 1.08 ± 0.20	0.16
L-PSSR,* s*^*−1*^	− 0.79 ± 0.19	− 0.76 ± 0.20	− 0.84 ± 0.17	0.02*
R-PDSR,* s*^*−1*^	− 2.65 ± 0.64	− 2.57 ± 0.56	− 2.74 ± 0.72	0.41
C-PDSR,* s*^*−1*^	1.39 ± 0.28	1.32 ± 0.24	1.48 ± 0.30	0.02*
L-PDSR,* s*^*−1*^	0.93 ± 0.26	0.83 ± 0.24	1.04 ± 0.25	< 0.001*
MR-based abdominal measures, mean ± SD				
H-PDFF, *%*	5.0 ± 4.8	7.7 ± 5.3	2.1 ± 0.5	< 0.001*
P-PDFF, *%*	3.6 ± 2.9	5.2 ± 3.1	1.8 ± 0.9	< 0.001*
SAT, *cm*^*2*^	168.1 ± 100.0	251.5 ± 60.3	76.7 ± 29.1	< 0.001*
VAT, *cm*^*2*^	75.6 ± 53.9	114.7 ± 46.8	32.6 ± 12.8	< 0.001*
Hepatic shear stiffness, *kPa*	2.35 ± 0.36	2.41 ± 0.37	2.29 ± 0.35	0.23

*ALT* alanine aminotransferase, *AST* aspartate aminotransferase, *BMI* body mass index, *BP* blood pressure, *b.p.m* beats per minute, *C-PDSR* circumferential peak diastolic strain rate, *C-PS* circumferential peak strain, *C-PSSR* circumferential peak systolic strain rate, *EAT* epicardial adipose tissue, *HDL-C* high-density lipoprotein cholesterol, *H-PDFF* hepatic proton density fat fraction, *IQR* interquartile range, *LDL-C* low-density lipoprotein cholesterol, *L-PDSR* longitudinal peak diastolic strain rate, *L-PS* longitudinal peak strain, *L-PSSR* longitudinal peak systolic strain rate, *LV* left ventricular, *LVEDV* left ventricular end-diastolic volume; *LVEF* left ventricular ejection fraction, *LVESV* left ventricular end-systolic volume, *LVM* left ventricular mass, *LVMT* left ventricular myocardial thickness, *MetS* metabolic syndrome, *MRE* magnetic resonance elastography, *NA* not applicable, *P-PDFF* pancreatic proton density fat fraction; *R-PDSR* radial peak diastolic strain rate, *R-PS* radial peak strain, *R-PSSR* radial peak systolic strain rate, *SAT* subcutaneous adipose tissue, *SD* standard deviation, *SV* stroke volume, *TC* total cholesterol, *TG* triglycerides, *VAT* visceral adipose tissue

Significant results are in bold

^*^The results were significant at a significance level of 0.05

### Comparisons of anthropometry, laboratory, and MR measures between subjects with obesity and healthy controls

BMI, waist and hip circumferences, systolic and diastolic BP, ALT, TC, TG, LDL-C, fasting and 2-h serum glucose in subjects with obesity were all significantly higher than those in healthy controls (all *p* ≤ 0.001), HDL-C in subjects with obesity was significantly lower than that in healthy controls (*p* < 0.001) (Table 2).

**Fig. 2:**
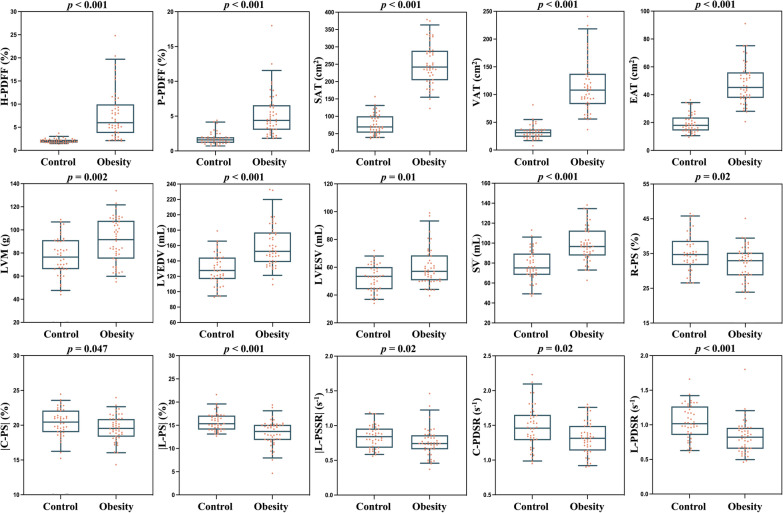
Boxplots of MR-based measures showing significant differences between obesity and healthy controls. Subjects with obesity had significantly higher H-PDFF, P-PDFF, SAT, VAT, EAT, LVM, LVEDV, LVESV, SV, and had significantly lower R-PS, |C-PS|, |L-PS|, |L-PSSR|, C-PDSR and L-PDSR than healthy controls

**Fig. 3:**
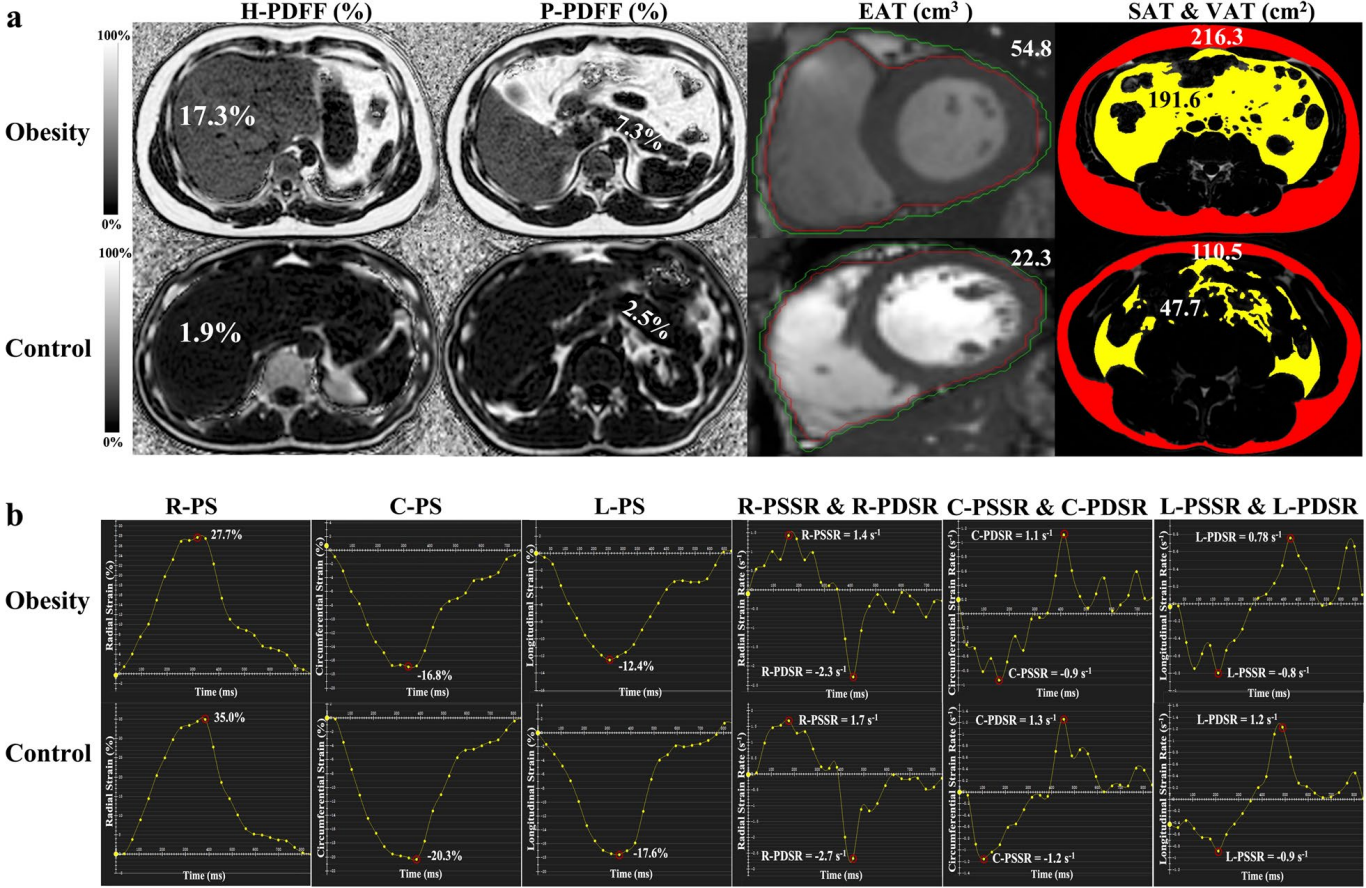
Examples of (**a**) MR images and (**b**) LV myocardial strain parameters in a 39-year-old man with obesity and in a healthy 34-year-old man

Compared to healthy controls, subjects with obesity had higher EAT, LVM, LVEDV, LVESV, SV, H-PDFF, P-PDFF, SAT, and VAT (all *p* ≤ 0.01), and lower R-PS, |C-PS|, |L-PS|, |L-PSSR|, C-PDSR, and L-PDSR (all *p* < 0.05) (Figs. 2, 3; Table 2). LVMT, LVEF, R-PSSR, |C-PSSR|, |R-PDSR|, and hepatic shear stiffness did not show significant differences between subjects with obesity and healthy controls (all *p* ≥ 0.10) (Table 2).

### Associations of H-PDFF with LV measures

**Fig. 4:**
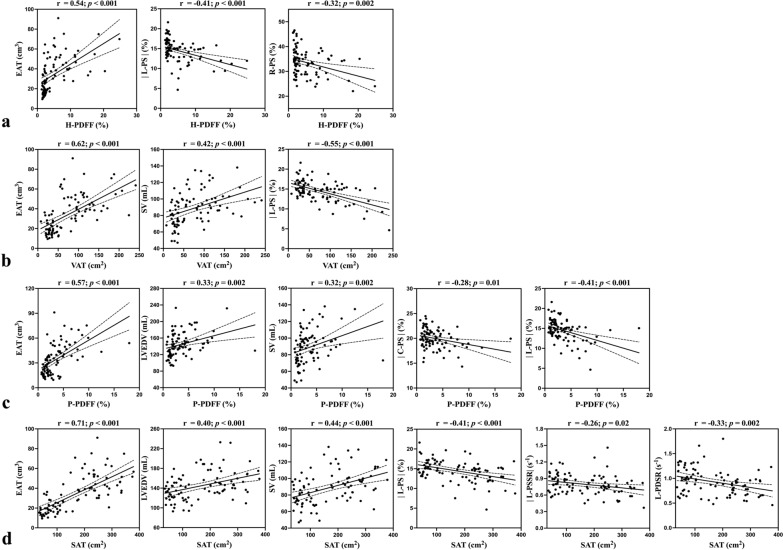
Scatterplots of significant associations in multivariable analyses (Model 1 and Model 2) in the over-all cohort. Pearson correlation coefficients (r) and *p* values are provided for the correlation between (**a**) H-PDFF, (**c**) P-PDFF, (**d**) SAT, (**b**) VAT and MR-based cardiac measures. The solid line indicates the line of best fit by using the least squares method, and the dotted line shows the 95% confidence interval

In the over-all cohort, H-PDFF was positively correlated with EAT, LVM, LVEDV, and SV (r = 0.24 to 0.54, all *p* ≤ 0.02), and was inversely correlated with R-PS, |C-PS|, |L-PS|, |L-PSSR|, |R-PDSR|, C-PDSR, and L-PDSR (rho = − 0.21 to − 0.41, all *p* < 0.05) in univariate analyses. The associations of H-PDFF with EAT, R-PS, |L-PS| persisted in both models (all *p* ≤ 0.04) (Fig. 4).

**Fig. 5:**
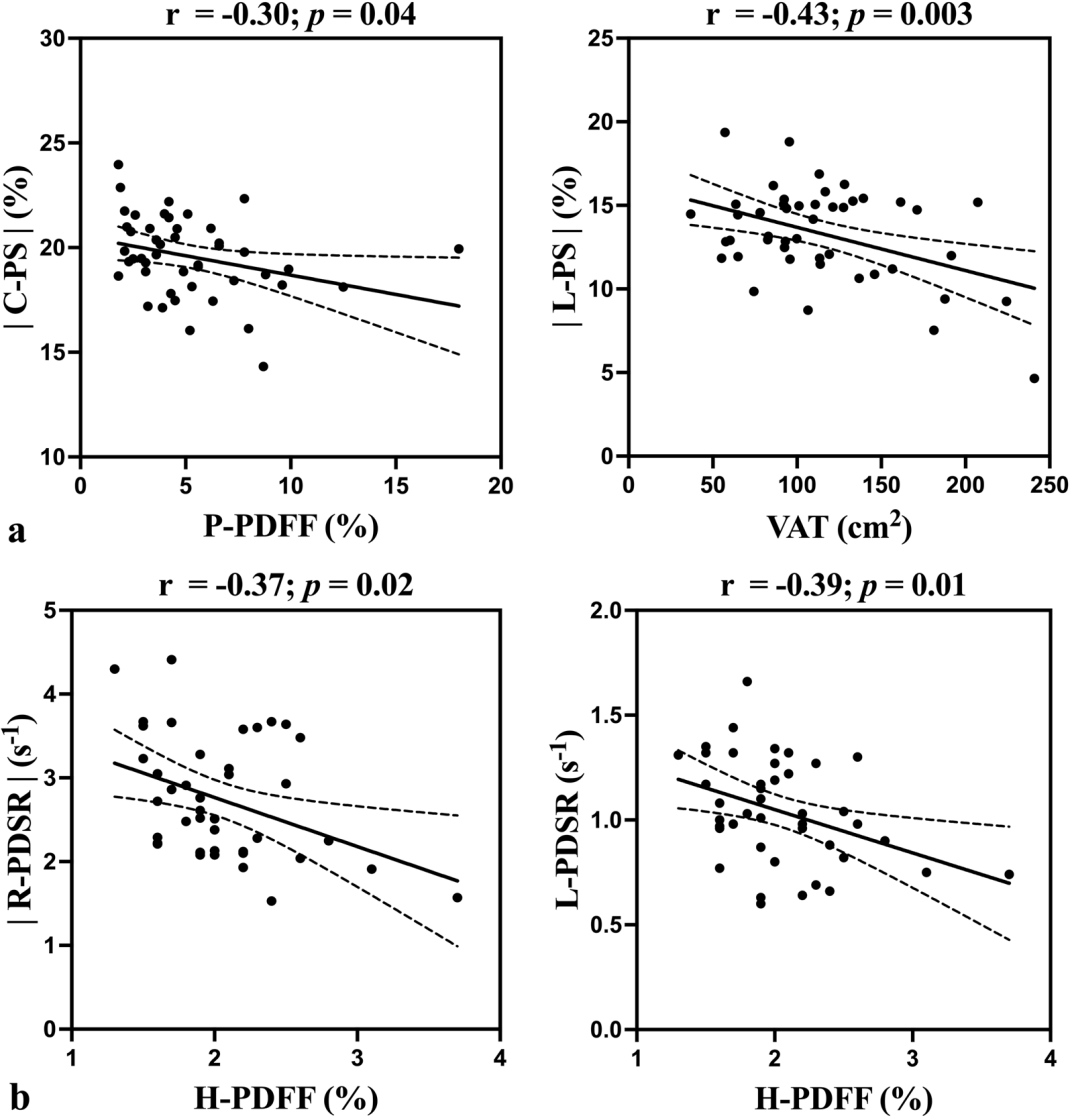
Scatterplots of significant associations in multivariable analyses (Model 1 and Model 2) in (**a**) subjects with obesity and (**b**) healthy controls. Pearson correlation coefficients (r) and *p* values are provided for the correlation between H-PDFF, P-PDFF, VAT and LV global myocardial strain parameters. The solid line indicates the line of best fit by using the least squares method, and the dotted line shows the 95% confidence interval

In subjects with obesity, there were negative correlations between H-PDFF and |R-PDSR|, C-PDSR in univariate analyses (r = − 0.30 and − 0.33, *p* = 0.045 and 0.03, respectively). Age, sex and MetS did not affect these associations (*p* = 0.046 and 0.04, respectively). However, the associations were not significant in Model 2 (*p* = 0.27 and 0.36, respectively). In healthy controls, |C-PS|, |R-PDSR|, and L-PDSR decreased significantly with increasing H-PDFF in univariate analyses (r = − 0.36 to − 0.39, all *p* ≤ 0.02). The associations between H-PDFF and |R-PDSR|, L-PDSR remained significant in both models (all *p* ≤ 0.04) (Fig. 5).

### Associations of P-PDFF with LV measures

In the over-all cohort, EAT, LVM, LVEDV, LVESV, and SV increased significantly with increasing P-PDFF (r = 0.24 to 0.57, all *p* ≤ 0.02); R-PS, |C-PS|, |L-PS|, C-PDSR, and L-PDSR decreased significantly with increasing P-PDFF (r = − 0.22 to − 0.41, all *p* ≤ 0.04) in univariate analyses. The associations between P-PDFF and EAT, LVEDV, SV, |C-PS|, and |L-PS| remained significant in both models (all *p* ≤ 0.02) (Fig. 4).

In subjects with obesity, P-PDFF was inversely correlated with C-PS in univariate analysis (r = − 0.30, *p* = 0.04), which was not affected by age, sex, hypertension, TG, HDL-C, prediabetes, or MetS (*p* = 0.01) (Fig. 5). In healthy controls, there was no significant association between P-PDFF and any LV measure in univariate analyses (all *p* > 0.05).

### Associations of SAT with LV measures

In the over-all cohort, there were positive correlations between SAT and EAT, LVM, LVEF, LVEDV and SV (r = 0.22 to 0.71, all *p* ≤ 0.04), and negative correlations between SAT and R-PS, |L-PS|, |L-PSSR| and L-PDSR (r = − 0.23 to − 0.41, all *p* ≤ 0.04) in univariate analyses. The associations between SAT and EAT, LVEDV, SV, |L-PS|, |L-PSSR| and L-PDSR persisted in both models (all *p* ≤ 0.04) (Fig. 4).

In subjects with obesity, there was no significant association between SAT and any LV measure in univariate analyses (all *p* ≥ 0.08). In healthy controls, there was a negative correlation between SAT and LVMT in univariate analysis (r = − 0.35, *p* = 0.02), while it did not remain significant in either model (*p* = 0.86 and 0.89, respectively).

### Associations of VAT with LV measures

In the over-all cohort, VAT was positively correlated with EAT, LVM, LVMT, LVEDV, LVESV, and SV (r = 0.23 to 0.62, all *p* ≤ 0.03), and was negatively correlated with |C-PS|, |L-PS|, C-PDSR, and L-PDSR (r = − 0.23 to − 0.55, all *p* ≤ 0.03) in univariate analyses. VAT was associated with EAT, SV, |L-PS| in both models (all *p* ≤ 0.02) (Fig. 4).

In subjects with obesity, there was a positive correlation between VAT and LVMT (r = 0.36, *p* = 0.02), and there were negative correlations between VAT and |L-PS|, C-PDSR, and L-PDSR (r = − 0.38 to − 0.44, all *p* ≤ 0.01) in univariate analyses. Only |L-PS| decreased with increasing VAT in both models (*p* = 0.002 and < 0.001, respectively) (Fig. 5). In healthy controls, VAT was inversely correlated with C-PS in univariate analysis (r = − 0.31, *p* = 0.049), which was of no significance in either model (*p* = 0.18 and 0.17, respectively).

### Associations of hepatic shear stiffness with LV measures

In univariate analyses, there was a negative correlation between hepatic shear stiffness and |L-PS| in the over-all cohort (rho = − 0.25, *p* = 0.02). There were positive correlations between hepatic shear stiffness and LVM, LVMT, and SV in subjects with obesity in univariate analyses (rho = 0.31 to 0.34, all *p* ≤ 0.04); however, they did not remain significant in multivariable analyses (*p* ≥ 0.05). In healthy controls, there was no significant association between hepatic shear stiffness and any cardiac measure in univariate analyses (all *p* ≥ 0.08).

Results of univariate analyses and multivariable regression tests for the associations between MR-based abdominal and cardiac measures are shown in Tables 3, 4, 5 and 6. Results of reproducibility analyses are shown in the Additional file 1.

**Table 3:** Results of Associations between MR-based Abdominal and Cardiac Measures using Pearson Correlation Tests

	H-PDFF (r, *p*)			P-PDFF (r, *p*)			SAT (r, *p*)			VAT (r, *p*)			Hepatic shear stiffness (r, *p*)		
	All	Obesity	Control	All	Obesity	Control	All	Obesity	Control	All	Obesity	Control	All	Obesity	Control
EAT	**0.54,** ** < 0.001**^**a**^	0.16, 0.29	0.03, 0.85	**0.57,** ** < 0.001**^**a**^	0.29, 0.05	− 0.30, 0.06	**0.71,** ** < 0.001**^**a**^	0.07, 0.67	0.23, 0.14	**0.62,** ** < 0.001**^**a**^	0.10, 0.52	− 0.22, 0.16	0.13, 0.23	0.04, 0.80	− 0.06, 0.70
LVM	**0.24,** **0.02**^**a**^	0.06, 0.70	0.03, 0.83	**0.25,** **0.02**^**a**^	0.06, 0.70	0.09, 0.58	**0.22,** **0.04**^**a**^	− 0.15, 0.31	− 0.29, 0.06	**0.39,** ** < 0.001**^**a**^	0.22, 0.14	0.27, 0.08	0.21, 0.06	**0.34,** **0.02**^**a**^	− 0.07, 0.68
LVMT	0.20, 0.06	0.19, 0.20	0.18, 0.25	0.13, 0.23	0.08, 0.61	0.06, 0.72	0.03, 0.78	− 0.11, 0.45	− **0.35,** **0.02**^**a**^	**0.29,** **0.01**^**a**^	**0.36,** **0.02**^**a**^	0.19, 0.23	0.14, 0.19	**0.31,** **0.04**^**a**^	− 0.12, 0.44
LVEF	0.12, 0.27	− 0.01, 0.93	0.06, 0.73	0.06, 0.61	− 0.14, 0.37	0.04, 0.81	**0.26,** **0.01**^**a**^	0.24, 0.11	0.05, 0.74	0.17, 0.11	0.05, 0.76	− 0.06, 0.71	0.01, 0.95	0.03, 0.86	− 0.09, 0.59
LVEDV	**0.26,** **0.02**^**a**^	− 0.12, 0.43	0.11, 0.43	**0.33,** **0.002**^**a**^	− 0.01, 0.96	0.23, 0.15	**0.40,** ** < 0.001**^**a**^	− 0.15, 0.33	− 0.20, 0.20	**0.40,** ** < 0.001**^**a**^	-0.07, 0.63	0.30, 0.06	0.20, 0.06	0.27, 0.07	− 0.06, 0.72
LVESV	0.17, 0.12	− 0.05, 0.74	0.03, 0.87	**0.24,** **0.02**^**a**^	0.06, 0.71	0.13, 0.43	0.21, 0.05	− 0.19, 0.20	− 0.16, 0.30	**0.23,** **0.03**^**a**^	-0.10, 0.53	0.27, 0.08	0.17, 0.12	0.16, 0.30	0.07, 0.66
SV	**0.26,** **0.02**^**a**^	− 0.15, 0.34	0.12, 0.43	**0.32,** **0.002**^**a**^	− 0.05, 0.72	0.22, 0.16	**0.44,** ** < 0.001**^**a**^	− 0.08, 0.60	− 0.17, 0.30	**0.42,** ** < 0.001**^**a**^	-0.04, 0.80	0.22, 0.16	0.18, 0.09	**0.31,** **0.04**^**a**^	− 0.12, 0.46
R-PS	− **0.32,** **0.002**^**a**^	− 0.28, 0.06	− 0.07, 0.67	− **0.22,** **0.04**^**a**^	− 0.16, 0.29	0.20, 0.21	− **0.23,** **0.04**^**a**^	0.08, 0.62	0.05, 0.75	− 0.16, 0.13	0.09, 0.53	0.16, 0.30	− 0.16, 0.13	− 0.10, 0.52	− 0.15, 0.36
|C-PS|	− **0.28,** **0.01**^**a**^	− 0.29 0.09	− **0.36** **0.02**^**a**^	− **0.28,** **0.01**^**a**^	− **0.30,** **0.04**^**a**^	0.07, 0.66	− 0.15, 0.16	− 0.01, 0.96	0.24, 0.12	− **0.23,** **0.03**^**a**^	-0.08, 0.59	− **0.31,** **0.049**^**a**^	-0.14, 0.22	-0.21, 0.17	0.01, 0.94
|L-PS|	− **0.41,** ** < 0.001**^**a**^	− 0.23, 0.13	− 0.07, 0.66	− **0.41,** ** < 0.001**^**a**^	− 0.22, 0.14	− 0.14, 0.38	− **0.41,** ** < 0.001**^**a**^	− 0.06, 0.69	0.07, 0.68	− **0.55,** ** < 0.001**^**a**^	**-0.43,** **0.003**^**a**^	− 0.05, 0.76	− **0.25,** **0.02**^**a**^	− 0.28, 0.07	− 0.09, 0.56
R-PSSR	− 0.11, 0.31	− 0.03, 0.85	− 0.12, 0.44	− 0.12, 0.27	− 0.02, 0.88	0.04, 0.82	− 0.19, 0.08	− 0.13, 0.38	0.08, 0.61	− 0.02, 0.85	0.20, 0.19	0.06, 0.73	− 0.19, 0.08	− 0.02, 0.88	− 0.26, 0.10
|C-PSSR|	− 0.09, 0.43	0.08, 0.60	− 0.04, 0.80	− 0.12, 0.28	− 0.07, 0.65	0.16, 0.32	− 0.21, 0.05	− 0.23, 0.12	0.11, 0.48	− 0.08, 0.47	0.21, 0.17	0.02, 0.92	− 0.14, 0.19	0.11, 0.50	− 0.28, 0.08
|L-PSSR|	− **0.21,** **0.047**^**a**^	− 0.13, 0.39	− 0.04, 0.79	-0.11, 0.31	− 0.01, 0.95	0.20, 0.21	− **0.26,** **0.02**^**a**^	− 0.16, 0.28	− 0.09, 0.58	− 0.19, 0.07	-0.06, 0.71	0.02, 0.92	− 0.19, 0.08	− 0.09, 0.56	− 0.26, 0.10
|R-PDSR|	− **0.25,** **0.02**^**a**^	− **0.30,** **0.045**^**a**^	− **0.37,** **0.02**^**a**^	− 0.08, 0.48	− 0.01, 0.96	0.03, 0.83	− 0.01, 0.95	0.26, 0.08	0.19, 0.23	− 0.19, 0.07	-0.16, 0.28	− 0.22, 0.16	0.11, 0.30	0.11, 0.48	0.17, 0.29
C-PDSR	− **0.33,** **0.002**^**a**^	− **0.33,** **0.03**^**a**^	− 0.04, 0.78	− **0.31,** **0.004**^**a**^	− 0.24, 0.11	− 0.19, 0.22	− 0.17, 0.12	0.15, 0.31	0.23, 0.14	− **0.39,** ** < 0.001**^**a**^	**-0.44,** **0.002**^**a**^	− 0.10, 0.55	0.004, 0.97	− 0.10, 0.54	0.18, 0.25
L-PDSR	− **0.37,** ** < 0.001**^**a**^	− 0.22, 0.15	− **0.39,** **0.01**^**a**^	− **0.32,** **0.002**^**a**^	− 0.11, 0.47	− 0.22, 0.16	− **0.33,** **0.002**^**a**^	− 0.002, 0.99	0.21, 0.18	− **0.47,** ** < 0.001**^**a**^	**-0.38,** **0.01**^**a**^	− 0.12, 0.44	− 0.13, 0.22	− 0.14, 0.36	− 0.01, 0.96

*EAT* epicardial adipose tissue, *H-PDFF* hepatic proton density fat fraction, *L-PDSR* longitudinal peak diastolic strain rate, *L-PS* longitudinal peak strain, *L-PSSR* longitudinal peak systolic strain rate, *LV* left ventricular; *LVEDV* left ventricular end-diastolic volume, *LVEF* left ventricular ejection fraction, *LVESV* left ventricular end-systolic volume, *LVM* left ventricular mass; *LVMT* left ventricular myocardial thickness, *P-PDFF* pancreatic proton density fat fraction, *R-PDSR* radial peak diastolic strain rate; *R-PS* radial peak strain, *R-PSSR* radial peak systolic strain rate, *SAT* subcutaneous adipose tissue, *SV* stroke volume, *VAT* visceral adipose tissue

Significant results are in bold

^a^The results were significant at a significance level of 0.05

**Table 4:** Associations between MR-based Abdominal and Cardiac Measures by Multivariable Regression in Over-all Cohort

		H-PDFF (β [95%CI], *p*)	P-PDFF (β [95%CI], *p*)	SAT (β [95%CI], *p*)	VAT (β [95%CI], *p*)	Hepatic shear stiffness (β [95%CI], *p*)
EAT	*Model 1*	**1.71 (0.93, 2.48), < 0.001**^**a**^	**3.34 (2.21, 4.47), < 0.001**^**a**^	**0.12 (0.09, 0.16), < 0.001**^**a**^	**0.23 (0.15, 0.31), < 0.001**^**a**^	–
	*Model 2*	**0.95 (0.07, 1.83), 0.04**^**a**^	**2.71 (1.66, 3.75), < 0.00**^**a**^	**0.10 (0.07, 0.13), < 0.001**^**a**^	**0.14 (0.04, 0.24), 0.01**^**a**^	–
LVM	*Model 1*	0.51 (− 0.24, 1.26), 0.18	**1.50 (0.35, 2.64), 0.01**^**a**^	**0.05 (0.02, 0.09), 0.003**^**a**^	**0.12 (0.04, 0.20), 0.003**^**a**^	–
	*Model 2*	0.45 (− 0.44, 1.34), 0.32	1.15 (− 0.02, 2.32), 0.05	0.04 (− 0.002, 0.08), 0.07	0.10 (− 0.001, 0.21), 0.05	–
LVMT	*Model 1*	–	–	–	0.002 (− 0.001, 0.01), 0.21	–
	*Model 2*	–	–	–	0.001 (− 0.003, 0.01), 0.68	–
LVEF	*Model 1*	–	–	0.01 (− 0.001, 0.02), 0.08	–	–
	*Model 2*	–	–	0.01 (0.000, 0.03), 0.05	–	–
LVEDV	*Model 1*	0.79 (− 0.45, 2.02), 0.21	**3.08 (1.25, 4.91), 0.001**^**a**^	**0.13 (0.08, 0.18), < 0.001**^**a**^	**0.18 (0.06, 0.31), 0.01**^**a**^	–
	*Model 2*	0.77 (− 0.68, 2.22), 0.29	**2.49 (0.63, 4.36), 0.01**^**a**^	**0.12 (0.06, 0.18), < 0.001**^**a**^	0.15 (− 0.02, 0.32), 0.08	–
LVESV	*Model 1*	–	**1.05 (0.17, 1.94), 0.02**^**a**^	–	0.05 (− 0.02, 0.11), 0.14	–
	*Model 2*	–	0.83 (− 0.09, 1.74), 0.08	–	0.00 (− 0.08, 0.08), 0.99	–
SV	*Model 1*	0.48 (− 0.40, 1.36), 0.28	**2.03 (0.71, 3.34), 0.003**^**a**^	**0.09 (0.06, 0.13), < 0.001**^**a**^	**0.14 (0.05, 0.23), 0.003**^**a**^	–
	*Model 2*	0.62 (− 0.44, 1.68), 0.25	**1.66 (0.29, 3.04), 0.02**^**a**^	**0.09 (0.05, 0.13), < 0.001**^**a**^	**0.15 (0.03, 0.27), 0.02**^**a**^	–
R-PS	*Model 1*	− **0.41 (**− **0.67, **− **0.15), 0.002**^**a**^	**-0.51 (-0.92, -0.09), 0.02**^**a**^	− **0.01 (**− **0.03, -0.001), 0.03**^**a**^	–	–
	*Model 2*	− **0.40 (**− **0.73, **− **0.07), 0.02**^**a**^	− 0.44 (− 0.89, − 0.001), 0.05	− 0.01 (− 0.02, 0.01), 0.23	–	–
|C-PS|	*Model 1*	− **0.11 (**− **0.21, -0.01), 0.03**^**a**^	− **0.19 (**− **0.34, -0.04), 0.01**^**a**^	–	− 0.01 (− 0.02, 0.002), 0.13	–
	*Model 2*	− 0.12 (− 0.24, 0.01), 0.06	− **0.19 (**− **0.35, **− **0.03), 0.02**^**a**^	–	− 0.01 (− 0.02, 0.01), 0.23	–
|L-PS|	*Model 1*	− **0.18 (**− **0.30, -0.06), 0.01**^**a**^	− **0.34 (**− **0.53, **− **0.15), 0.001**^**a**^	− **0.01 (**− **0.02, **− **0.006), < 0.001**^**a**^	− **0.03 (**− **0.04, **− **0.02), < 0.001**^**a**^	− 1.48 (− 2.98, 0.01), 0.05
	*Model 2*	− **0.17 (**− **0.32, **− **0.02), 0.03**^**a**^	− **0.29 (**− **0.49, **− **0.10), 0.004**^**a**^	− **0.01 (**− **0.02, **− **0.003), 0.01**^**a**^	− **0.03 **− **0.05, **− **0.01), < 0.001**^**a**^	− 1.39 (− 2.83, 0.05), 0.06
|L-PSSR|	*Model 1*	− **0.01 (**− **0.19, 0.00), 0.04**^**a**^	–	− **0.001 (**− **0.001, 0.00), 0.01**^**a**^	–	–
	*Model 2*	− 0.01 (− 0.02, 0.001), 0.06	–	− **0.001 (**− **0.001, 0.00), 0.04**^**a**^	–	–
|R-PDSR|	*Model 1*	− **0.04 (**− **0.07, -0.01), 0.02**^**a**^	–	–	–	–
	*Model 2*	-0.03 (-0.07, 0.01), 0.18	–	–	–	–
C-PDSR	*Model 1*	− **0.02 (**− **0.03, **− **0.002), 0.02**^**a**^	− 0.02 (− 0.04, 0.002), 0.08	–	− **0.001 (**− **0.003, 0.00), 0.049**^**a**^	–
	*Model 2*	− 0.01 (− 0.02, 0.01), 0.32	− 0.01 (− 0.04, 0.01), 0.19	–	− 0.001 (− 0.003, 0.001), 0.50	–
L-PDSR	*Model 1*	− **0.02 (**− **0.03, **− **0.01), 0.01**^**a**^	− **0.02 (**− **0.04, **− **0.001), 0.04**^**a**^	− **0.001 (**− **0.002, 0.00), 0.001**^**a**^	− **0.002 (**− **0.003, **− **0.001), 0.002**^**a**^	–
	*Model 2*	− 0.01 (− 0.03, 0.003), 0.13	− 0.02 (− 0.04, 0.004), 0.11	− **0.001 (**− **0.001, 0.00), 0.01**^**a**^	− 0.001 (− 0.003, 0.00), 0.10	–

*CI* confidence interval, *C-PDSR* circumferential peak diastolic strain rate, *C-PS* circumferential peak strain, *H-PDFF* hepatic proton density fat fraction, *L-PDSR* longitudinal peak diastolic strain rate, *L-PS* longitudinal peak strain, *LV* left ventricular; *LVM* left ventricular mass, *LVMT* left ventricular myocardial thickness, *P-PDFF* pancreatic proton density fat fraction, *R-PDSR* radial peak diastolic strain rate, *SV* stroke volume, *VAT* visceral adipose tissue

Significant results are in bold

^a^The results were significant at a significance level of 0.05

**Table 5:** Associations between MR-based Abdominal and Cardiac Measures by Multivariable Regression in Obesity Subgroup

		H-PDFF (β [95%CI], *P*)	P-PDFF (β [95%CI], *P*)	VAT (β [95%CI], *P*)	Hepatic Shear Stiffness (β [95%CI], *P*)
LVM	*Model 1*	–	–		5.43 (− 7.51, 18.36), 0.40
	*Model 2*	–	–		6.19 (− 7.72, 19.61), 0.36
LVMT	*Model 1*	–	–	0.002 (− 0.003, 0.01), 0.49	0.07 (− 0.42, 0.56), 0.78
	*Model 2*	–	–	0.001 (− 0.004, 0.01), 0.60	0.17 (− 0.35, 0.69), 0.51
SV	*Model 1*	–	–		7.73 (− 2.62, 18.08), 0.14
	*Model 2*	–	–		7.30 (− 6.95, 21.54), 0.31
|C-PS|	*Model 1*	–	− **0.25 (**− **0.44, **− **0.07), 0.01**^**a**^		–
	*Model 2*	–	− **0.29 (**− **0.48, **− **0.09), 0.01**^**a**^		–
|L-PS|	*Model 1*	–	–	− **0.04 (**− **0.06, -0.01), 0.002**^**a**^	–
	*Model 2*	–	–	− **0.05 (**− **0.07, -0.02), < 0.001**^**a**^	–
|R-PDSR|	*Model 1*	− **0.03 (**− **0.06, **− **0.001), 0.046**^**a**^	–		–
	*Model 2*	− 0.02 (− 0.06, 0.02), 0.27	–		–
C-PDSR	*Model 1*	− **0.01 (**− **0.03, **− **0.001), 0.04**^**a**^	–	− **0.002 (**− **0.004, 0.00), 0.03**^**a**^	–
	*Model 2*	− 0.01 (− 0.02, 0.01), 0.36	–	− 0.001 (− 0.003, 0.001), 0.26	–
L-PDSR	*Model 1*	–	–	− 0.002 (− 0.004, 0.00), 0.05	–
	*Model 2*	–	–	− 0.002 (− 0.004, 0.001), 0.15	–

*CI* confidence interval, *C-PDSR* circumferential peak diastolic strain rate, *C-PS* circumferential peak strain, *H-PDFF* hepatic proton density fat fraction, *L-PDSR* longitudinal peak diastolic strain rate, *L-PS* longitudinal peak strain, *LV* left ventricular, *LVM* left ventricular mass, *LVMT* left ventricular myocardial thickness, *P-PDFF* pancreatic proton density fat fraction, *R-PDSR* radial peak diastolic strain rate; *SV* stroke volume, *VAT* visceral adipose tissue

Significant results are in bold

^a^The results were significant at a significance level of 0.05

**Table 6:** Associations between MR-based Abdominal and Cardiac Measures by Multivariable Regression in Healthy Controls

		H-PDFF (β [95%CI], *p*)	SAT (β [95%CI], *p*)	VAT (β [95%CI], *p*)
LVMT	*Model 1*	–	0.001 (− 0.01, 0.01), 0.86	–
	*Model 2*	–	0.001 (− 0.01, 0.01), 0.89	–
|C-PS|	*Model 1*	− **1.30 (**− **2.58, -0.01), 0.048**^**a**^	–	− 0.03 (− 0.08, − -0.02), 0.18
	*Model 2*	− 1.31 (− 2.76, 0.14), 0.08	–	− 0.04 (− 0.10, 0.02), 0.17
|R-PDSR|	*Model 1*	− **0.50 (**− **0.98, -0.02), 0.04**^**a**^	–	–
	*Model 2*	− **0.61 (**− **1.14, -0.08), 0.03**^**a**^	–	–
L-PDSR	*Model 1*	− **0.21 (**− **0.36, **− **0.07), 0.01**^**a**^	–	–
	*Model 2*	− **0.23 (**− **0.40, **− **0.06), 0.01**^**a**^	–	–

*CI* confidence interval, *C-PS* circumferential peak strain, *H-PDFF* hepatic proton density fat fraction, *L-PDSR* longitudinal peak diastolic strain rate, *LV* left ventricular, *LVMT* left ventricular myocardial thickness, *R-PDSR* radial peak diastolic strain rate, *SAT* subcutaneous adipose tissue, *VAT* visceral adipose tissue

Significant results are in bold

^a^The results were significant at a significance level of 0.05

## Discussion

In this study, we mainly explored the associations between MR-based abdominal adiposity, hepatic shear stiffness, and subclinical alterations of LV geometry and function in adults free of overt CVD. We found that despite the LVEF being within the normal range, (1) subjects with obesity exhibited alterations in LV geometry compared to healthy controls, and P-PDFF, SAT and VAT were positively associated with LVEDV and SV independent of MetS in the over-all cohort; (2) subjects with obesity had subclinical LV dysfunction manifested by decreased myocardial strain, and H-PDFF, P-PDFF, SAT and VAT were inversely associated with LV global myocardial strain parameters independent of MetS in the over-all cohort; (3) In the obesity subgroup, increasing P-PDFF and VAT were independently associated with decreasing C-PS and L-PS; and (4) hepatic shear stiffness showed no independent association with EAT or subclinical LV remodeling.

### Association between abdominal adiposity and LV geometry

In our study, subjects with obesity, who had higher visceral fat depots (EAT and VAT) and ectopic fat deposition (H-PDFF and P-PDFF), had higher LVEDV, LVESV, and SV than healthy controls, which was similarly observed by prior studies using echocardiography or cardiovascular MR [20–22]. It may be explained by the fact that individuals with obesity require the higher metabolism of both lean and visceral adipose tissue, generating higher volume overload and cardiac output [23]. In addition, the increased LVEDV and SV with P-PDFF, SAT, and VAT may be due to increased metabolism of excess ectopic fat and adipose tissue. Unexpectedly, LVM, LVEDV, and SV increased significantly with H-PDFF in univariate analyses, while the association was of no significance after adjusting for age, sex, and the diagnosis of MetS/MetS-related covariates. One of the plausible reasons may be that this association was mainly attributed to MetS. Future studies are warranted.

### Association of ectopic fat deposition in liver and pancreas with subclinical LV dysfunction

Although the LVEF of all subjects was within the normal range and no significant difference existed between the obesity and control subgroups, subjects with obesity had impaired LV systolic and diastolic function compared to healthy controls, manifested by lower R-PS, |C-PS|, |L-PS|, |L-PSSR|, C-PDSR, and L-PDSR. It indicated that subclinical LV dysfunction had occurred in individuals with obesity before LVEF was reduced. Moreover, global myocardial peak strain decreased with increasing H-PDFF (with R-PS and L-PS) and P-PDFF (with C-PS and L-PS) independent of MetS in the over-all cohort, indicating that higher ectopic fat in the liver and pancreas was associated with worse subclinical LV function. Similarly, Levelt et al. [9] found that individuals with obesity and type 2 diabetes exhibited impairment in peak systolic circumferential strain and diastolic strain rates, and hepatic triglyceride measured by MR spectroscopy correlated negatively with the above two strain parameters in univariate analyses. It should be noted that multivariable analysis was not performed in their study, and only circumferential strain parameters were evaluated. In our study, not only circumferential but also radial and longitudinal strain parameters were assessed, which provided more information regarding the myocardial deformation in different directions. No article on the relationship between MR-based pancreatic steatosis and cardiac geometry or function was found through a thorough search of the relevant literature. The pancreas is a common location where ectopic fat deposits in obesity, which generates oxidative stress and inflammatory responses leading to myocardial injury through hyperglycemia induced by islet cell dysfunction [24, 25]. It could partially explain why higher P-PDFF was associated with impaired LV function in our study, which provides a possible positive signal for future research. The detailed mechanisms connecting these two entities need to be further studied.

### Association between abdominal SAT, VAT and subclinical LV dysfunction

In our study, higher VAT was independently associated with worse subclinical LV function manifested by lower |L-PS| in both the over-all cohort and obesity subgroup. Nevertheless, the inverse association between SAT and LV myocardial deformation observed in the over-all cohort was not shown in the obesity subgroup after adjusting for covariates. These findings suggest that VAT may play a more critical role in the connection with LV subclinical dysfunction than SAT in individuals with obesity. A prior study reported similar observations in subjects from the general population without a history of CVD, finding an independent association for VAT to measures of LV remodeling but not for SAT [7]. The underpinning mechanism may be that, compared to SAT, VAT produces more proinflammatory adipokines, such as adiponectin and leptin, leading to myocardial damage [26].

### Association of hepatic shear stiffness with EAT and LV remodeling

The present study did not find a significant association between hepatic shear stiffness and EAT; although there were significant associations of hepatic shear stiffness with LV geometry and function in univariate analyses, they were attenuated when taking account of age, sex, and MetS. Our findings are not in accordance with prior studies [12, 13]. Brouha et al. [12] found that liver fibrosis measured using MRE was independently associated with EAT in diabetic individuals without known CVD. Petta et al. [13] observed that a higher EAT was associated with the severity of histologic liver fibrosis in patients with NAFLD. One of the possible reasons for the inconsistency may be that the cohort compositions are not identical. In our study, subjects with obesity and healthy controls were recruited, and no subject had type 2 diabetes; whereas in their studies, all subjects had diabetes or NAFLD without including healthy controls. Thus, our cohorts tended to show a lower proportion of liver fibrosis than their studies, manifested by only six subjects with MRE-based liver fibrosis (hepatic shear stiffness ≥ 2.9 kPa [27]). Further studies are warranted to explore the effect of obesity or MetS on the association between liver fibrosis and cardiac geometry and function.

### Limitations

First, this is a single-center, cross-sectional study. Given that the design precludes inferring the causality of the observational interferences, it is unclear whether there would be a reversion of myocardial function with the reduction of visceral and ectopic fat depositions. Besides, it is a cross-sectional and exploratory study, and correction for multiple comparisons and the interdependencies of some cardiac MR measures, such as LVEF and LVEDV, were not taken into account. The given p-values are used for exploratory purposes, and the results with a p value < 0.05 mainly provide a possible positive signal for future research. Further longitudinal and multi-center studies with more sophisticated statistical methods are needed to corroborate our findings in the future. Second, our sample size, especially for the subjects with liver fibrosis, is relatively small; and no subject had diabetes. While on the other hand, our study inadvertently eliminated the potential effect of diabetes on the association between abdominal adiposity and cardiac geometry and function. A larger study population with sufficient subjects in diverse fibrosis stages and hyperglycemic states is required to assess the associations in various subgroups. Third, we utilized LV two- and four-chamber cine images in the long-axis view for the measurement of global strain parameters, consistent with some prior studies [28, 29]. Further studies embracing complete long-axis cine images (two-, three-, and four-chamber images) as described in other studies [30–32] may be warranted to confirm our findings. Finally, in order to better reduce the white coat effect, BP was measured after 15 min of rest. While it should be noted that some studies founded that automated office BP measures taken after 8 min of rest tended to be lower than daytime ambulatory BP [33]. Future studies in which BP is measured after a shorter period of rest (e.g., 5 min) may be warranted.

## Conclusions

In adults free of overt CVD, higher H-PDFF, P-PDFF, SAT, and VAT were associated with worse subclinical LV function, and LVEDV and SV increased with P-PDFF, SAT, and VAT independent of MetS. In individuals with obesity, VAT rather than SAT was associated with subclinical LV dysfunction. Hepatic shear stiffness showed no independent association with EAT or LV geometry or LV function. Our results suggest that ectopic fat depositions in the liver and pancreas and excess abdominal adipose tissue pose a risk of subclinical LV dysfunction beyond MetS-related CVD risk factors, and VAT may play a more considerable role as a risk factor for subclinical LV dysfunction than SAT in obesity. The mechanisms underpinning these associations and their longitudinal clinical implications need to be further investigated.

## Supplementary Information

**Additional file 1**: **Table S1**. Intra- and interobserver variability of cardiac and abdominal MR measures.

## Data Availability

The datasets generated and analyzed during the current study are available from the corresponding authors on reasonable request.

## Abbreviations

ALT: Alanine aminotransferase
AST: Aspartate aminotransferase
BMI: Body mass index
BP: Blood pressure
b.p.m: Beats per minute
bSSFP: Balanced steady-state free precession
BW: Bandwidth
C-PDSR: Circumferential peak diastolic strain rate
C-PS: Circumferential peak strain
C-PSSR: Circumferential peak systolic strain rate
CVD: Cardiovascular disease
EAT: Epicardial adipose tissue
ECG: Electrocardiogram
FA: Flip angle
FOV: Field of view
HDL-C: High-density lipoprotein cholesterol
H-PDFF: Hepatic proton density fat fraction
ICC: Intraclass correlation coefficient
IDEAL-IQ: Iterative decomposition of water and fat with echo asymmetry and least-squares estimation quantitation
LAVA-Flex: Liver acquisition with volume acceleration flex
LDL-C: Low-density lipoprotein cholesterol
LV: Left ventricular
L-PDSR: Longitudinal peak diastolic strain rate
L-PS: Longitudinal peak strain
L-PSSR: Longitudinal peak systolic strain rate
LV: Left ventricular
LVEDV: Left ventricular end-diastolic volume
LVEF: Left ventricular ejection fraction
LVESV: Left ventricular end-systolic volume
LVM: Left ventricular mass
LVMT: Left ventricular myocardial thickness
MetS: Metabolic syndrome
MR: Magnetic resonance
MRE: Magnetic resonance elastography
NEX: Number of excitations
OGTT: Oral glucose tolerance test
P-PDFF: Pancreatic proton density fat fraction
R-PDSR: Radial peak diastolic strain rate
R-PS: Radial peak strain
R-PSSR: Radial peak systolic strain rate
SAT: Subcutaneous adipose tissue
SE-EPI: Spin-echo echo-planar imaging
SV: Stroke volume
TC: Total cholesterol
TE: Echo time
TG: Triglycerides
TR: Repetition time
VAT: Visceral adipose tissue
