# Supplementary material for: Association of abdominal adiposity, hepatic shear stiffness with subclinical left-ventricular remodeling evaluated by magnetic resonance in adults free of overt cardiovascular diseases: a prospective study
Source: Cardiovasc Diabetol. 2023 Apr 29;22:99. doi: 10.1186/s12933-023-01828-1 (PMC10149007; doi:10.1186/s12933-023-01828-1)
Supplement: Supplementary file 1 — Additional file 1: Table S1. Intra- and interobserver variability of cardiac and abdominal MR measures. [file 12933_2023_1828_MOESM1_ESM.docx]

**Additional file**

**Cardiac MR analysis**

EAT volume was measured on the short-axis cine slices during the end-diastolic phase. The myoepicardial and parietal pericardial contours were manually delineated in each slice, extending from the mitral valve hinge down to the ventricular apex. Regions of high signal intensity between the myoepicardium and parietal pericardium were semi-automatically traced and calculated after the exclusion of blood vessels.

The LV geometry and global systolic function were measured on the short-axis cine images. The endo- and epicardial contours of the LV myocardium were manually traced during the end-diastolic and end-systolic phases.

LV global myocardial strain was assessed on the long-axis two- and four-chamber and short-axis cine images. These cine images were uploaded into the 3D tissue-tracking module of CVI 42 software, and the endo- and epicardial contours were then automatically delineated in each slice during the end-diastolic phase in all series, excluding the papillary muscles and moderator band. Manual adjustment of endo- and epicardial contours was conducted afterwards. LV strain reflects the degree of myocardial deformation from the initial length (L_0_; in end-diastole) to its maximum length (L; in end-systole) of the myocardium, and is calculated from the equation: myocardial strain = (L – L_0_)/L_0_. Strain rate represents the rate of shortening of length.

**Abdominal MR analysis**

To prevent the analyst from receiving feedback from PDFF maps that may bias the placement of the regions of interest (ROIs), ROIs were placed in the magnitude images initially and copied to the PDFF maps subsequently. The fifth echo of the magnitude images consistently yielding adequate anatomical delimitation were chose. One circular 1-cm radius ROI was placed in each of the nine Couinaud segments of liver, and a circular 0.5-cm radius ROI was placed in pancreatic head, body and tail respectively. PDFF values from these ROIs were averaged to obtain the whole-liver (H-PDFF) or whole-pancreas PDFF (P-PDFF) for each subject.

On axial fat images by LAVA-Flex sequence, the middle three slices at the third lumbar (L3) and fourth lumbar (L4) intervertebral space (L3–L4) level were collected to measure SAT and VAT area. The mean SAT and VAT areas were calculated for each subject.

Four axial slices centered over the portal vein bifurcation of MRE were acquired. On each of the four images, one free-form ROI was drawn in the largest possible area of the right hepatic lobe within the boundaries of the 95% confidence mask while remaining coherent shear waves and avoiding liver edges, large blood vessels, bile ducts and artifacts observed on magnitude images. The mean shear stiffness values from the four ROIs were estimated for each subject.

**Reproducibility**

The intra- and interobserver variability were calculated for the LV global myocardial strain parameters and abdominal MR measures among all participants. The intraobserver variability was measured twice by the same observer with 2-week interval. The interobserver variability were performed by two independent observers who were blinded to the results of the other’s measurements. Results of reproducibility analyses are shown in Table S1.

| **Table S1 Intra- and interobserver variability of cardiac and abdominal MR measures** | | | | | |
| --- | --- | --- | --- | --- | --- |
|  | **Intraobserver** | |  | **Interobserver** | |
|  | **ICC*** | **95% CI** |  | **ICC*** | **95% CI** |
| **Cardiac geometry** |  |  |  |  |  |
| LVM (g) | 0.989 | 0.977 – 0.999 |  | 0.970 | 0.944 – 0.988 |
| LVMT (mm) | 0.977 | 0.943 – 0.990 |  | 0.974 | 0.936 – 0.989 |
| LVEF (%) | 0.949 | 0.751 – 0.983 |  | 0.886 | 0.758 – 0.953 |
| LVEDV (mL) | 0.990 | 0.983 – 0.995 |  | 0.980 | 0.976 – 0.994 |
| LVESV (mL) | 0.978 | 0.968 – 0.997 |  | 0.958 | 0.859 – 0.972 |
| SV (mL) | 0.947 | 0.918 – 0.966 |  | 0.925 | 0.867 – 0.966 |
| **PS (%)** |  |  |  |  |  |
| Radial | 0.921 | 0.799 – 0.991 |  | 0.887 | 0.709 – 0.945 |
| Circumferential | 0.932 | 0.842 – 0.989 |  | 0.850 | 0.629 – 0.951 |
| Longitudinal | 0.900 | 0.727 – 0.950 |  | 0.838 | 0.631 – 0.931 |
| **PSSR (s^-1^)** |  |  |  |  |  |
| Radial | 0.880 | 0.766 – 0.956 |  | 0.874 | 0.674 – 0.929 |
| Circumferential | 0.902 | 0.799 – 0.980 |  | 0.901 | 0.854 – 0.981 |
| Longitudinal | 0.832 | 0.654 – 0.903 |  | 0.820 | 0.656 – 0.896 |
| **PDSR (s^-1^)** |  |  |  |  |  |
| Radial | 0.882 | 0.630 – 0.956 |  | 0.840 | 0.659 – 0.959 |
| Circumferential | 0.834 | 0.613 – 0.938 |  | 0.809 | 0.598 – 0.954 |
| Longitudinal | 0.868 | 0.632 – 0.936 |  | 0.819 | 0.605 – 0.950 |
| **EAT (cm^3^)** | 0.886 | 0.716 – 0.959 |  | 0.810 | 0.677 – 0.943 |
| **H-PDFF (%)** | 0.980 | 0.960 – 0.990 |  | 0.978 | 0.949 – 0.990 |
| **P-PDFF (%)** | 0.977 | 0.961 – 0.987 |  | 0.971 | 0.944 – 0.985 |
| **SAT** **(cm^2^)** | 0.984 | 0.966 – 0.993 |  | 0.982 | 0.964 – 0.991 |
| **VAT (cm^2^)** | 0.973 | 0.939 – 0.988 |  | 0.971 | 0.941 – 0.986 |
| **Hepatic shear stiffness (kPa)** | 0.986 | 0.970 – 0.994 |  | 0.962 | 0.923 – 0.981 |
| ***All *p* < 0.001.** *CI* confidence interval; *EAT* epicardial adipose tissue; *H-PDFF* hepatic proton density fat fraction; *ICC* intraclass correlation coefficient; *LVEDV* left ventricular end-diastolic volume; *LVEF* left ventricular ejection fraction; *LVESV* left ventricular end-systolic volume; *LVM* left ventricular mass; *LVMT* left ventricular myocardial thickness; *P-PDFF* pancreatic proton density fat fraction; *PDSR* peak diastolic strain rate; *PS* peak strain; *PSSR* peak systolic strain rate; *SAT* subcutaneous adipose tissue; *SV* stroke volume; *VAT* visceral adipose tissue | | | | | |
